# Ambipolar zinc-polyiodide electrolyte for a high-energy density aqueous redox flow battery

**DOI:** 10.1038/ncomms7303

**Published:** 2015-02-24

**Authors:** Bin Li, Zimin Nie, M. Vijayakumar, Guosheng Li, Jun Liu, Vincent Sprenkle, Wei Wang

**Affiliations:** 1 Energy and Environment Directorate, Pacific Northwest National Laboratory, PO Box 999, Richland, Washington 99352, USA

## Abstract

Redox flow batteries are receiving wide attention for electrochemical energy storage due to their unique architecture and advantages, but progress has so far been limited by their low energy density (~25 Wh l^−1^). Here we report a high-energy density aqueous zinc-polyiodide flow battery. Using the highly soluble iodide/triiodide redox couple, a discharge energy density of 167 Wh l^−1^ is demonstrated with a near-neutral 5.0 M ZnI_2_ electrolyte. Nuclear magnetic resonance study and density functional theory-based simulation along with flow test data indicate that the addition of an alcohol (ethanol) induces ligand formation between oxygen on the hydroxyl group and the zinc ions, which expands the stable electrolyte temperature window to from −20 to 50 °C, while ameliorating the zinc dendrite. With the high-energy density and its benign nature free from strong acids and corrosive components, zinc-polyiodide flow battery is a promising candidate for various energy storage applications.

## Abstract (web summary)

Conventional redox flow batteries have low energy densities. Here the authors present an aqueous redox flow battery with an ambipolar and bifunctional zinc-polyiodide electrolyte, which exhibits an energy density approaching to that of lithium ion batteries.

## Graphical abstract

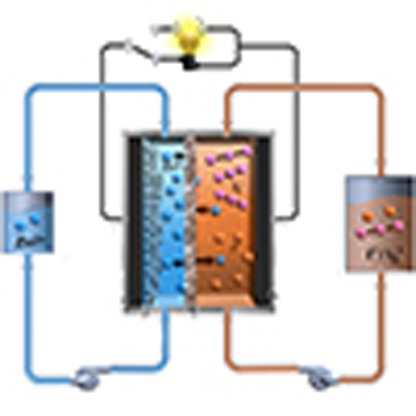

Unlike traditional batteries, flow-based electrochemical energy storage systems separate the energy storage and power generation by storing the electro-active species in externally flowing electrolytes (that is, the anolyte and catholyte), while maintaining the redox reactions at the electrode surface inside a stack^1^,^2^. This unique architecture permits the redox flow batteries (RFBs) to independently scale the power and/or energy—a characteristic advantage along with high safety coveted by the energy industry for intermittent renewable energy integration and other grid services^3^. The energy density (*E*, based on the electrolyte (that is, active materials) compositions and volumes) of a RFB is usually measured as the amount of energy stored per unit volume of electrolyte and can be described by the concentration of active redox species (*C*_a_) and voltage (*V)* in the form of equation (1):


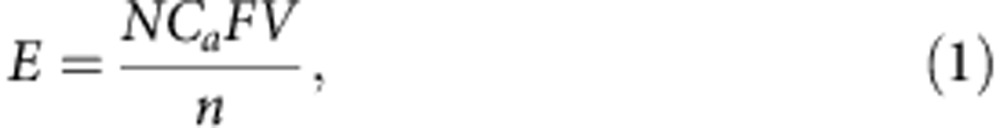


where *N* is the number of electrons involved in the redox reaction, *F* is the Faraday constant (26.8 Ah mol^−1^) and *n* is the number of electrolyte volumes contributing to redox reactions. An improvement in *E* requires maximization of both *C*_a_ and *V*, while minimizing *n.*

Despite continuous progress^3^,^4^, the energy density of traditional aqueous RFBs is considerably lower than that of low-end lithium (Li) ion batteries such as those with LiFePO_4_ cathodes (>223 Wh l^−1^ based on the cell level^5^). Traditional aqueous RFBs, such as all-vanadium (vanadium redox battery, VRB) and Fe/Cr systems, are generally limited to <25 Wh l^−1^ by both the low *V* owing to water electrolysis and the low *C*_a_ due to the relatively low solubility of the active species^6^,^7^,^8^. Although it is generally believed that the energy density is not a critical performance criterion for stationary applications, the low energy density of flow-based batteries has nevertheless largely excluded themselves from other energy storage market. Improvement in energy density is therefore a necessity to enable the flow battery for applications other than stationary energy storage. Although progress has been made to achieve high *V* value (>2.0 V) through a variety of non-aqueous RFB designs^3^, such as those based on all-organic active materials^9^ and redox non-innocent ligands^10^, the redox species concentration currently is limited to around 0.1 M or less resulting in an energy density <10 Wh l^−1^, even for higher cell voltages.

To design a high-energy density flow battery, the *n* value in equation (1) needs to be minimized to 1. Traditional RFB systems, such as the VRB and Fe/Cr RFBs, utilize different cationic redox couples at the anode and cathode half-cells. The value of *n* in those systems is equal to 2 considering the involvement of two liquid electrolyte volumes at both sides. Apparently, hybrid flow batteries, where one half-cell features a redox reaction involving a redox species that is not fully soluble (such as a metal electrode), are attractive. Although the presence of a metal electrode limits the complete dissociation of the energy and power in a hybrid flow battery, it is possible to achieve high energy density with this design when paired with an electrolyte possessing ambipolar characteristics, since the *n* value can be reduced to 1. In such electrolytes, cationic and anionic ions from a single soluble compound are both energy-bearing redox active species eliminating the need for non-active counter ions such as the Cl^−^ and SO_4_^2−^ commonly used in VRB and Fe/Cr RFB systems, while enable the flow system to use a negligible amount of electrolyte at one of the half-cells. In addition, under a given cell voltage (*V*), the *C*_a_ needs to be maximized to achieve a high energy density. Bifunctional electrolytes, in which at least one of the active species acts as the charge carrier, are thus particularly appealing due to the reduction in the requirement for a high-concentration supporting electrolyte, which can adversely affect the maximum concentration attainable for the active species. Traditional aqueous Zn/Br flow batteries (ZBB)^3^ and the recently developed non-aqueous Li–S and Li–iodine RFB^11^,^12^,^13^ follow the above design strategies. The energy densities of the ZBB and Li–S systems, however, are compromised by the limited achievable maximum concentrations of the soluble redox species (ZBB is ~65 Wh l^−1^ (refs 6, 8), while a Li–S RFB is ~108 Wh l^−1^ demonstrated by a static cell based on the soluble Li_2_S_y_ phases^12^). In a ZBB system, although the ZnBr_2_ is known to have high solubility^14^, the charged species Bromine (Br_2_) is insoluble in aqueous solution, which substantially limits the achievable energy density in ZBB system^15^ in addition to the high corrosiveness and health hazardous concerns of the Br^−^/Br_2_ redox couple. On the other hand, the Li–S and Li–iodine RFBs face numerous challenges, such as the high cost, flammable components, difficulty to scale-up and the low conductivity of the non-aqueous electrolyte and so on. It is therefore critical to identify new redox species with the high solubility and environmental friendliness to enable high-energy density aqueous flow battery with high safety.

On the basis of the above considerations, we demonstrate that the zinc-polyiodide electrolyte possesses the desired ambipolar and bifunctional characteristics, as well as the high solubility and benign nature, thus enabling a high-energy density aqueous hybrid RFB. Capitalizing on the high solubility of the I^−^/I_x_^−^ redox species, the zinc-polyiodide flow battery (ZIB) has a theoretical energy density of ~322 Wh l^−1^ at the solubility limit of ZnI_2_ in the water (4,500 g l^−1^, 7.0 M). We demonstrate here a discharge energy density of 166.7 Wh l^−1^ with 5.0 M ZnI_2_ electrolytes, nearly seven times that of the current aqueous RFBs (VRB: ~25 Wh l^−1^) and approaching the energy density of low-end LiFePO_4_ cathode-based Li ion batteries. Considering the substantial presence of non-active materials in the Li ion batteries (40–50 wt.% (ref. 16)), a high energy density flow battery is even more appealing free from the inactive and costly components, such as the tabs, current collector foils, package cases and electrode binders and so on. The ZIB system maintains the attractive architecture of flow battery designs with the favourable features of high safety and no hazardous materials/strong acids.

## Results

### Scheme of aqueous Zn–I flow batteries

**Figure 1:**
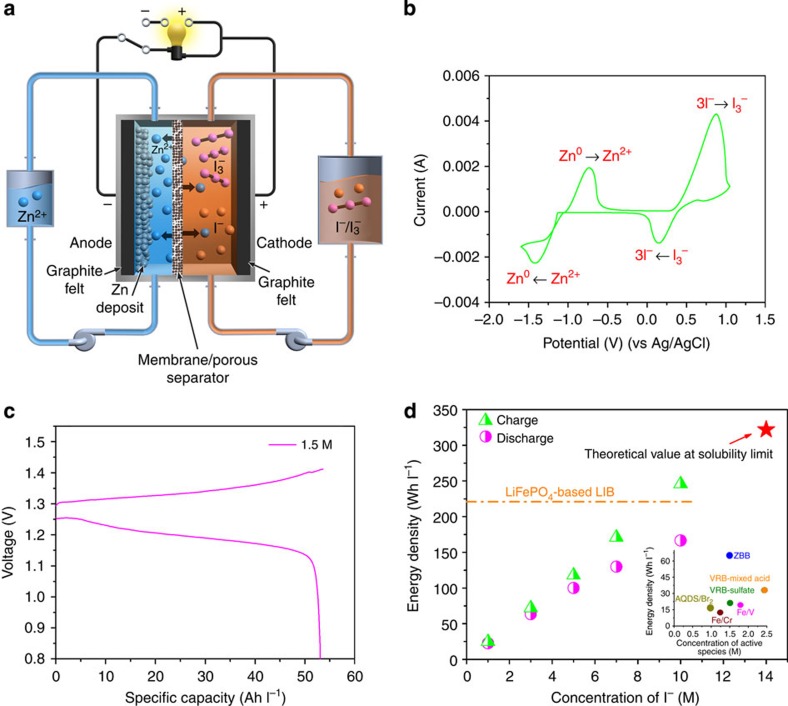
Zn–I RFB and its electrochemical performance. (**a**) Schematic representation of the proposed ZIB system. (**b**) CV of 0.085 M ZnI_2_ on a glassy carbon electrode at the scan rate of 50 mV s^−1^. (**c**) Typical charge–discharge curves at 1.5 M ZnI_2_ at a current density of 20 mA cm^−2^. (**d**) The charge and discharge energy densities as a function of the concentration of I^−^. The inset lists concentration versus energy density of several current aqueous RFB chemistries for comparison^3^,^6^,^7^,^8^.

The schematic of the proposed ZIB is shown in Fig. 1a. A cation exchange membrane (CEM) (Nafion) is sandwiched between two pristine graphite felts (GFs) electrodes, while the zinc iodide solutions are pumped through both half-cells. Although the Zn/Iodine redox couple has been previously proposed for a primary battery^17^, its application in liquid-based secondary RFBs is unfeasible because of the extreme low solubility of I_2_. However, the I_2_ solubility significantly increases with the presence of iodide due to the formation of polyiodide ions^18^:


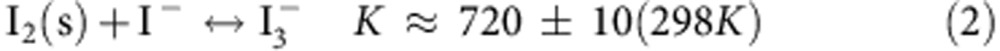


Triiodide ions have been reported as the predominant species in aqueous solution owing to the low stability of other higher order polyiodides^18^,^19^. The dominant redox reaction in the system is believed to be between I_3_^−^/I^−^, as shown in equation (3) because of the fast charge transfer rate of the I_3_^−^ reduction reaction^19^,^20^. A number of other redox reactions are potentially possible^18^,^20^. There are substantial potential separations, however, away from the I_3_^−^/I^−^ redox couple. A Zn-polyiodide RFB is thus constructed based on the following redox reactions:


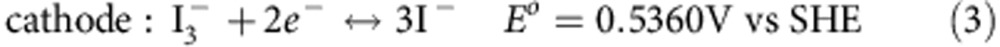



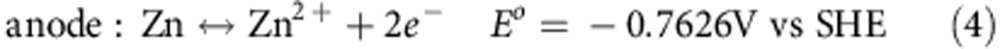



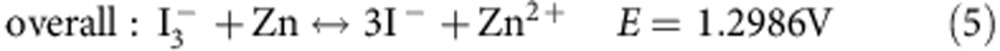


The aqueous electrolyte at both the sides is composed of ZnI_2_ salt dissolved in water. During the charge process, metallic zinc is electroplated on the negative electrode, while polyiodide ions are formed in solution at the positive side. On discharge the reverse processes occur. The bifunctional zinc ions act as both charge carriers transported through the membrane and the active species at the negative half-cell.

### Electrochemical performance of aqueous Zn–I flow batteries

A cyclic voltammogram of 0.085 M electrolyte of ZnI_2_ on a glassy carbon working electrode in the range of −1.6 to 1.0 V (versus Ag/AgCl) at the scan rate of 50 mV s^−1^ shows the two pairs of redox reaction peaks (Fig. 1b), corresponding to the redox reaction of Zn/Zn^2+^ at the negative and I_3_^−^/I^−^ at the positive half-cells, respectively. Owing to the nearly neutral nature of the solutions (pH values: 3–4 at 0% state of charge, SOC) and the low potential of the I_3_^−^/I^−^ redox couple (0.536 V versus SHE), neither H_2_ nor O_2_ evolution peaks are observed in Fig. 1b throughout the whole-scan range.

The cell evaluation was conducted under a constant current density of 20 mA cm^−2^. The charge/discharge experiment is designed to have both upper voltage and capacity limits, allowing all of the iodide ions at the cathode side to be oxidized to triiodide ions, while avoiding the formation of excess I_2_ (to prevent precipitation). The electrochemical performance of the ZIB system as a function of ZnI_2_ concentrations is shown in Table 1 (Current densities are 20 for 0.5–3.5 M ZnI_2_ electrolytes, and 5 mA cm^−2^ for 0.5–3.5 M and 5.0 M ZnI_2_ electrolyte, respectively.) and the voltage profiles are shown in Supplementary Fig. 1. Figure 1c presents a typical charge–discharge voltage curve of 1.5 M ZnI_2_ electrolyte at the current density of 20 mA cm^−2^. It can be seen from Table 1 and Supplementary Fig. 1 that the open-circuit voltage decreases from 1.430 to 1.270 V with increasing concentration of ZnI_2_ from 0.5 to 3.5 M, while the coulombic efficiency (CE) remains nearly constant at ~99%, which indicates the high cation selectivity of the membrane used in the flow cell. As a CEM, the Nafion membrane used in the current study contributes to the high CE values due to the negligible crossover of active I^−^ or I_3_^−^ species owing to their anion nature and large ionic radius. The negligible anion crossover is corroborated with an X-ray photoelectron spectroscopy analysis of the used Nafion membrane in ZIB (Supplementary Fig. 2; Supplementary Methods), which shows no evidence of I while a large amount of Zn was identified. In traditional RFBs, such as VRB and Fe/Cr RFBs, protons are often used as charge carriers, usually introduced through various acids in the supporting electrolytes. When a CEM is used, the simultaneous transport of the active cation ions (such as V and Fe ions) together with protons gives rise to the problematic crossover issue often observed in those systems, leading to a low CE (<96%) and energy losses from the subsequent self-discharge reactions following crossover. The ambipolar and bifunctional designs of the ZIB utilize zinc ions as both the redox active species and charge carriers, eliminating the competition of transport between these two. Together with the high selectivity of the Nafion membrane against iodide/triiodide anions, the ZIB delivers a much improved CE value. In addition, the energy efficiency (EE) values decrease from 90.9 to 76.0% as the concentration of ZnI_2_ increases from 0.5 to 3.5 M, which is mainly attributed to the decreasing voltage efficiency (VE) due to the increasing electrolyte resistance arising from increasing concentration of the electrolytes. The electrochemical performance, including the CE, VE and EE values, of the ZIB flow cell with a 3.5 M ZnI_2_ electrolyte cycled under various charge/discharge current densities is displayed in Supplementary Fig. 3a and Supplementary Table 1. Considering the intermittent nature of many grid-scale energy storage applications, a low self-discharge is essential for high performance. A delayed discharge test is therefore performed on a 2.5 M ZnI_2_ electrolyte after different rest durations following the fully charge. As shown in Supplementary Fig. 3b, the discharge voltage curves (rest after fully charge for 30 s and 1 day) are overlapped with each other, which suggest the excellent stability of the ZIB system and polyiodide ions even at the fully charged condition.

**Figure 2:**
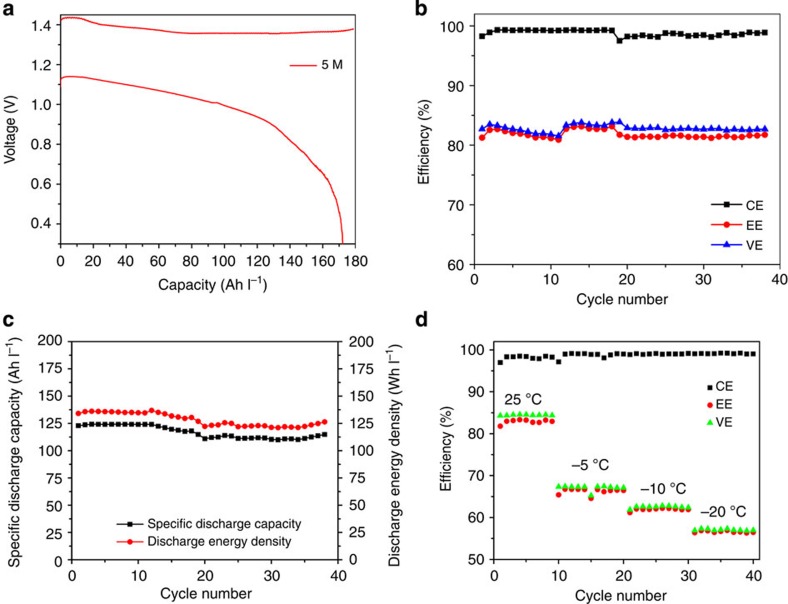
Cycling performances of the Zn–I flow batteries. (**a**) Charge/discharge curves for the cell with 5.0 M ZnI_2_ and Nafion 115 as membrane operated at the current density of 5 mA cm^−2^. (**b**) Cycling performances for efficiencies of the cell with 3.5 M ZnI_2_ and Nafion 115 as membrane under the current density of 10 mA cm^−2^. (**c**) Cycling performances for discharge energy densities and capacities of the cell with 3.5 M ZnI_2_ and Nafion 115 as membrane under the current density of 10 mA cm^−2^. (**d**) Efficiencies of the cell with (2.5 M ZnI_2_+10 vol% EtOH) and Nafion 115 as membrane tested under the current density of 10 mA cm^−2^ at different temperatures.

Detailed explanations for the calculation of the energy density of the ZIBs are provided in the Supplementary Note 1. On charge or discharge, the electroplating or electrodissolution of zinc at the anode side is inevitably accompanied by the transport of zinc ions from one side to another for charge balance. The amount of zinc ions in the anolytes remains constant. As a result, in essence, the specific energy density of a ZIB flow cell is solely dependent on the concentration of the catholyte. The volume of the effective active species contributing to the energy output of the flow cell is therefore defined as the volume of the catholyte (*n*=1 in equation (1)). The experimental charge and discharge energy densities of the ZIB flow cell as a function of the concentration of I^−^ are plotted and compared with other flow battery chemistries in Fig. 1d. The charge and discharge energy densities increase with increasing concentration of the iodide ions. The theoretical energy density for the ZIB could reach 322 Wh l^−1^ at the solubility limit of ZnI_2_ in water (~14 M I^−^). The inset in Fig. 1d displays the actual energy density versus concentration of the active species of several aqueous RFB systems for comparison^3^,^6^,^7^,^8^. The charge/discharge voltage curves of the flow cell with a 5.0 M ZnI_2_ electrolyte are shown in Fig. 2a. As shown in Fig. 1d, based on the discharge energy density that measures the ultimate capability of the system to deliver useful energy, the ZIB flow cell at 5.0 M ZnI_2_ electrolyte could reach 166.7 Wh l^−1^, which is the highest energy density value achieved under real flow condition in any flow battery systems reported thus far. The cycling performance of the ZIB flow battery are evaluated with a 3.5 M ZnI_2_ electrolyte with a ZIB flow cell equipped with a Nafion 115 membrane under a current density of 10 mA cm^−2^. The cycling efficiency and capacity behaviour are shown in Fig. 2b,c. No obvious efficiency and capacity decay were observed demonstrating the high energy retention (>99%) over 40 cycles, which suggest that negligible side reactions occur during the charge/discharge reactions.

The theoretical capacity and the corresponding 100% SOC are defined as when all of the iodide ions at the cathode half-cell are fully converted to triiodide ions (I_3_^−^; Supplementary Note 1). As displayed in Fig. 2b,c, excellent cycling performance was obtained when the SOC was limited to 100% during charge. Continuous charging over the 100% SOC will result in an actual charge capacity exceeding the calculated theoretical value as shown in Supplementary Fig. 4, suggesting the existence of I_2_ or other polyiodide species, such as I_5_^−^ and I_7_^−^ (ref. 18). The cycling, however, is not sustainable under these conditions. Significant capacity decay was observed starting from the second cycle. After the disassembly of the cell, I_2_ precipitation was identified on the surface of GFs by Raman spectroscopy^21^, when the SOC was over 100%, as shown in Supplementary Fig. 5a,b. It is therefore necessary to investigate the chemistry of electrolytes at different SOC states to better understand the polyiodide chemistry in the ZIB system during the electrochemical charge/discharge processes.

### Catholyte Raman study

**Figure 3:**
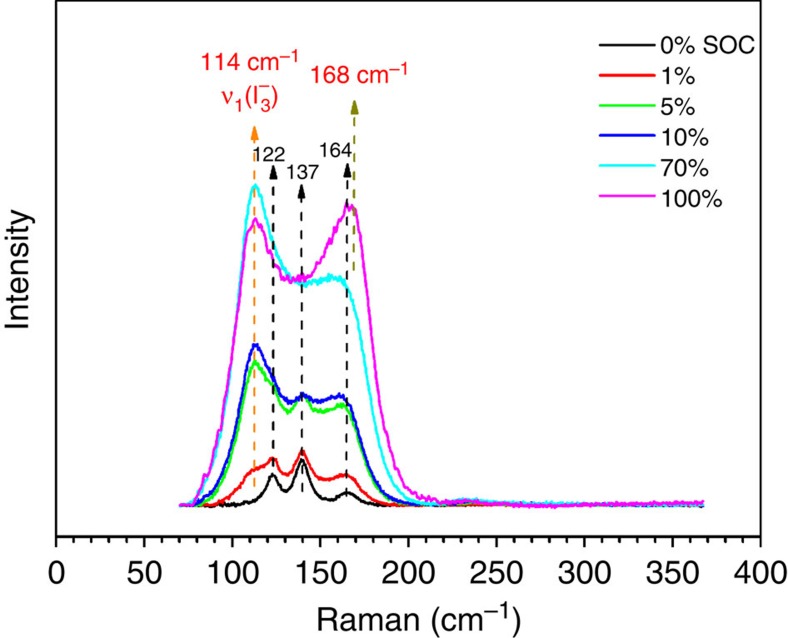
Raman study. Raman spectra of catholytes at different state-of-charges (SOCs) from 0 to 100% SOC.

Figure 3 shows the Raman spectra of the catholyte samples obtained at different SOCs (that is, 0, 1, 5, 10, 70 and 100%). The Raman spectrum of the catholyte at 0% SOC was characterized by three distinctive bands at 122, 137 and 164 cm^−1^. The three Raman bands observed are in good agreement with previous works reported in the literature^22^. These bands have been assigned to the symmetric stretching *v*_1_(*A*_1_) vibrational modes of [ZnI_4_]^2−^, [ZnI_3_]^−^ and [ZnI_2_] complexes existing in the aqueous phase. On charging, an additional Raman band at 114 cm^−1^ starts to appear and its intensity increases as the SOC increases. Evidently, this band is associated with the species, such as polyiodides, generated during the charging process. To confirm the existence of polyiodides, Raman spectra of two standard salt samples with tetrabutylammonium cations (TBA-I_3_ and TBA-I_5_) were obtained (Supplementary Fig. 6a,b; Supplementary Methods). The observed representative Raman bands for the triiodide (I_3_^−^) and pentaiodide (I_5_^−^) anions are at 110 and 168 cm^−1^, respectively, and have been assigned to the *v*_1_(symmetric stretch) of I_3_^−^ and *v*_1_(outer symmetric stretch) of I_5_^−^ in the literature^18^. Therefore, the band at 114 cm^−1^ could be attributed to the *v*_1_ mode of I_3_^−^. Raman study was also carried out on the catholyte during discharge. As shown in Supplementary Fig. 6c, Raman spectra of catholytes at the same SOC during the charge and discharge processes are nearly identical. Particularly, there is no significant difference between the fully discharged (0% SOC) catholyte and the original electrolyte, which suggests that there is no residual I_3_^−^ after fully discharge. The Raman study data together with the high CE value of the flow cell test indicate the excellent reversibility of the system.

It is interesting to note that the Raman spectrum of the 100% SOC sample has two different peak shapes when compared with the Raman spectrum of the 70% SOC sample: (1) decreased intensity for the band at 114 cm^−1^ belonging to I_3_^−^ and (2) significantly increased intensity for the band at 168 cm^−1^. The assignment of the Raman band at 168 cm^−1^ appears to be quite challenging, since it is not completely resolved. One possible explanation is to attribute this to the formation of I_5_^−^ species. The overlapped Raman spectra at higher SOCs (100, 120 and 125%) in Supplementary Fig. 7, however, show that the composition of the solutions hardly changes after the precipitate (I_2_) was removed from the solutions, suggesting that the increasing higher order polyiodide ions become unstable in water with increasing SOC (over 100%). Such polyiodides likely decompose to I_2_ and iodide^18^,^23^. More extensive studies will be performed to determine the possible existence of I_5_^−^ or other polyiodides in catholytes at higher SOC.

### Solution chemistry of the ZnI_2_ electrolyte

The stability of the electrolyte is of critical importance for practical operation, as this not only dictates the system energy density, but also the stable operational window in terms of temperature and SOC range. The stability of the liquid ZnI_2_ electrolyte was thoroughly investigated through both off-line static and on-line cycling test. At high temperature (50 °C), no precipitation was observed with off-line electrolytes at various SOCs, and for both the half-cells over long-term cycling (10 days) with concentrations of ZnI_2_ as high as 3.5 M (Supplementary Fig. 8; Supplementary Table 2). The stability does appear to be an issue, however, for the fully charged catholytes at low temperature. A precipitate was found at 0 °C for the cell with 2.5 and 3.5 M ZnI_2_ electrolytes at 100% SOC at the positive side after 10 days of storage (Supplementary Table 2). This was identified to be I_2_ by Raman spectroscopy (Supplementary Fig. 5c)^21^.

**Figure 4:**
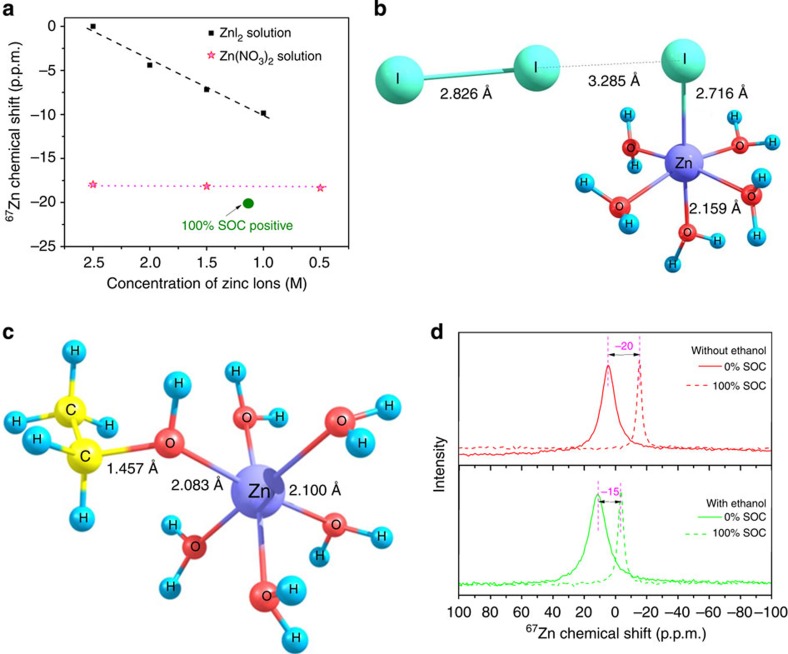
NMR and DFT studies of ZnI_2_ catholyte. (**a**) Chemical shift of ^67^Zn NMR for both standard Zn(NO_3_)_2_ and ZnI_2_ solutions at different zinc ion concentrations. The parent 2.5 M ZnI_2_ aqueous solution is used as reference (*d*_iso_=0 p.p.m.). DFT-optimized molecular structure of the (**b**) triiodide-complexed zinc cation and (**c**) EtOH-complexed zinc cation formed in the catholyte during the charging process. (**d**) ^67^Zn NMR peaks of the pristine and fully charged ZnI_2_ catholytes with and without EtOH. The lines represent pristine samples and dashes represent charged catholytes (at 100% SOC).

The I_2_ precipitation at low temperature is possibly caused by the shift of the equilibrium in equation (2), which is suspected due to the influence of the metal counter cation (that is, zinc ions). The dynamic interactions between the zinc and iodide species were therefore investigated by nuclear magnetic resonance (NMR) study and density functional theory (DFT)-based simulation. ^67^Zn NMR substantiates the complexation between the zinc ions and triiodide. The chemical shift (that is, peak position) under different charge states can shed light into the evolution of the molecular species in the ZIB electrolytes. To exclude the influence of the zinc ion concentration on the chemical shift, ^67^Zn NMR spectra for both aqueous Zn(NO_3_)_2_ and ZnI_2_ solutions at different zinc ion concentrations were measured (Fig. 4a). For the aqueous Zn(NO_3_)_2_ solutions, there was a monotonous chemical shift (~0.2 p.p.m./1 M) with decreasing concentration of zinc ions due to common hexaaqua-solvated zinc ions (that is, [Zn^2+^·6H_2_O]^2+^). For aqueous ZnI_2_ solutions, the different types of complexion of the zinc ions and iodide (Fig. 3; ref. 22) might induce chemical shifts towards a lower frequency with a reduction of the zinc ion concentration. However, on charge, as mentioned above, the zinc ion acting as a charge carrier together with water will transfer from the positive to negative side, which leads to the reduction of the electrolyte volume (Supplementary Fig. 9) and zinc ion concentration. At 100% SOC, the concentration of zinc ions was decreased to 1.14 M, but, as displayed in Fig. 4a, a significant chemical shift towards lower frequency (~20 p.p.m.) takes place, suggesting the formation of triiodide during the charging process that might chemically interact with the zinc cation by sharing electron density. To further analyse this possibility, we derived the molecular structure of the proposed [Zn.I_3_. 5H_2_O]^+^ complex using DFT calculations (Fig. 4b), the existence of which is confirmed by a mass spectroscopy analysis (Supplementary Fig. 10; Supplementary Methods). Moreover, we analysed the potential energy surface under the iodide-complexed molecular environment (that is, 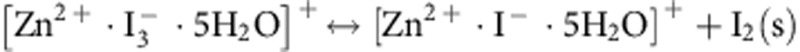) using DFT calculations. The lower energy barrier (ca. 0.2 eV) for the triiodide dissociation under zinc-complexed form can lead to the formation of molecular iodine (I_2_) that is known to have poor solubility in aqueous solutions, and thus result in precipitate formation from the catholyte at low temperatures (<0 °C).

As discussed above, the precipitation initiates from the formation of [Zn.I_3_. 5H_2_O]^+^ at the fully charged catholytes. Hence, controlling the complexing of the metal ions (Zn^2+^) is critical to avoid the I_2_ precipitation. A plausible strategy is to introduce another species, which is energetically more favourable for coordination with the zinc ions, thereby modifying the interactions between the zinc and triiodide ions. Alcohol is known to complex with zinc ions due to the presence of the oxygen lone-pair electrons, and thus could serve as an optimal additive to provide counter species 24. The influence of the ethanol (EtOH) as an additive on the stability of the catholytes was therefore investigated by NMR spectroscopy, along with DFT-based computational simulation studies. DFT calculations show that the oxygen in EtOH can bind with the zinc cations by replacing a water molecule from its primary solvation shell similar to the triiodide complexation discussed earlier (Fig. 4c). Figure 4d shows the ^67^Zn NMR of the catholyte at different charge states with and without a 10 vol% addition of EtOH. As shown in Fig. 4d, the solution with EtOH shows obvious line broadening effects relative to the solution without EtOH at 0% SOC, which indicates the possible existence of EtOH-complexed zinc cations. Moreover, the fully charged catholyte solution (100% SOC) with EtOH has smaller chemical shifts than for the solution without EtOH, implying that the EtOH-complexed zinc cations might partially hinder the formation of [Zn·I_3_· 5H_2_O]^+^ species. Hence, the triiodide dissociation and subsequent precipitation reaction might be mitigated due to the direct interaction of EtOH with the zinc cation. The introduction of organic additives (such as EtOH in our case) was found to significantly improve the stability of the catholytes at lower temperature. As shown in Supplementary Table 2, the positive side electrolytes with 3.5 M ZnI_2_ at 100% SOC can be stabilized (no precipitation) down to −20 °C on the addition of a 25 vol% of EtOH. As shown in Fig. 2d, a ZIB flow cell with a 2.5 M ZnI_2_ electrolyte with a 10 vol% of EtOH can be operated at a temperature as low as −20 °C. Other alcohols, such as ethylene glycol, are found to have a similar effect on stabilizing the triiodide electrolytes (Supplementary Table 2). Because of the addition of EtOH, the temperature window of the ZIB system becomes much wider than that of traditional VRBs (−5 to 50 °C), which shows a greater potential for ZIB to be adopted around the world with widely different climates. It is also worth noting that the addition of alcohol (EtOH) will decrease the conductivity of the electrolyte, which will then lead to a reduced VE due to a higher polarization from the electrolyte resistance as shown in Supplementary Fig. 11. The content of the alcohol therefore has to be optimized to achieve best performance.

The addition of EtOH and subsequent coordination formation between the oxygen atoms and zinc ions are also found to contribute to the zinc dendrite mitigation. Zinc dendritic growth is one of the major challenges for zinc-based batteries^25^. The flowing electrolyte environment in the ZIB reduces the dendrite growth rate. As shown in Supplementary Fig. 12, compared with a static ZIB (no electrolyte flow), the ZIB flow battery tested with the same electrolyte and current density, but with 100 ml min^−1^ of flowing electrolyte, demonstrated zinc dendrite sizes approximately four times smaller than those for the static cell (Supplementary Methods). Under the same experimental conditions, the addition of EtOH in the ZnI_2_ electrolyte also contributes to the mitigation of the zinc dendritic growth rendering a zinc surface with finer grain as shown in Supplementary Fig. 13. The additive-induced dendrite suppression process is believed to originate from the EtOH coordination with the zinc ions, which enhances the plating overpotential, thus lowering the plating exchange current density resulting in a smoother zinc surface^25^. Other inhibitors for zinc dendrite growth are currently being investigated.

## Discussion

In summary, we present here a successful demonstration of the high-energy density aqueous RFB system (zinc/polyiodide) with ambipolar and bifunctional electrolytes (ZnI_2_). The energy density is approaching the low end of that of Li ion battery. And it is proved experimentally and theoretically that the addition of alcohols into electrolytes could effectively stabilize the cathode electrolyte at lower temperature and ameliorate zinc dendrite growth at the anode because of ligand formation between oxygen on the hydroxyl group and the zinc ions. Besides, as compared with commercial VRBs and ZBBs, the ZIB provides much more design latitude in the choice and development of alternative membranes and additives because of the absence of highly oxidative V^5+^ and Br_2_ (ref. 26). Similar to the Fe/Cr and Fe/V RFB systems, a low-cost hydrocarbon CEM or porous separator can potentially be applied in the ZIB, thus replacing the expensive Nafion membrane, making the ZIB system more attractive from a cost prospective^27^. In addition, the architecture of the ZIB flow battery is compatible with traditional ZBB systems with less corrosiveness and can be readily scale-up. Together with its high energy density and high safety, the ZIB flow battery is a very promising candidate for various energy storage applications.

## Methods

### Preparation of lab-scale zinc-polyiodide flow cells

The makeup of a single cell was described in detail in previously published papers^28^. In each half-cell, porous GFs (SGL Carbon Group, Germany) with apparent areas of 40 cm^2^ served as the electrodes and gold-coated copper plates as current collectors. The commercially available CEMs (Nafion, Dupont, DE, USA) with different thicknesses were used as the membranes. Around 2 mm deep space between the anode surface and membrane is reserved for the electrodeposition of zinc metal. The original electrolytes with different concentrations were prepared by dissolving appropriate ZnI_2_ (Fisher Scientific, 98%) in deionized water (with or without EtOH) at room temperature. The single cell was connected to two glass cylinders to measure the volume changes of electrolytes during the charge/discharge process.

### Flow cell test

The electrochemical performance of the flow cell was carried out using a potentiostat/galvanostat (Arbin Instrument, USA) within a fixed voltage window between 0.3 and 1.5 V under a constant current mode operated under current densities ranging from 5 to 30 mA cm^−2^. The upper limit of the charge process was determined by both voltage (1.5 V) and theoretical capacity. The calculation of theoretical capacity is explained in detail in the supporting information. Seventy-five microlitres of electrolyte in the positive half-cell was pumped at a flow rate of 50 or 100 ml min^−1^ through a peristaltic pump. Due to the characteristics of the Zn–I flow battery, the negative half-cell does not require equal volume of the electrolyte as the positive half-cell. A small amount of electrolyte however was maintained in the negative half-cell and pumped at the same flow rate.

### Characterization

A cyclic voltammogram test was conducted in a three-electrode cell using a CHI660C workstation (CH Instruments, USA). A platinum wire, glassy carbon and Ag/AgCl electrode were used as the counter, working and reference electrodes, respectively. Testing was performed from −1.6 to 1.0 V versus Ag/AgCl reference electrode in water solutions of 0.085 M ZnI_2_ at a scan rate of 50 mV s^−1^.

To identify the chemistry of the catholytes, the original catholytes (2.0 M ZnI_2_) were charged with different SOCs. Then, Raman spectroscopy measurements were performed on an inverted microscope (Nikon Eclipse Ti) coupled with Raman spectroscopic system (Horiba Jobin Yvon). Red He–Ne laser (632.8 nm) was used as an excitation laser source.

The ^67^Zn NMR measurements were performed using a Varian 500 Inova spectrometer (B_0_=11.1 T and ^67^Zn Larmor frequency is 31.2 MHz). The quantitative ^67^Zn NMR spectra were recorded with single-pulse measurements at room temperature using 5-mm NMR tubes with a Varian liquid probe. The parent 2.5 M ZnI_2_ aqueous solution is used as reference (*δ*_iso_=0 p.p.m.) to effectively monitor the chemical shift evolution with the charge/discharge process. Aqueous ZnI_2_ and Zn(NO_3_)_2_ (Sigma-Aldrich, 99%) solutions with different concentrations were prepared and characterized by ^67^Zn NMR for comparison. Quantum chemistry calculations were carried out using the Amsterdam Density Functional (2013) program^29^. The hybrid-GGA-based Becke, three-parameter, Lee-Yang-Parr (B3LYP) DFT function and TZ2P (triple Z, 2 polarization functions, all electron) basis set along with relativistic zero-order regular approximation function is used. All basis sets and preceding calculations were done with the Slater type functional implemented in the Amsterdam Density Functional program for both geometry optimization and frequencies calculation. All the molecular structure optimizations were carried out using in-build COSMO solvation model with water as solvent. Other characterization methods are described in Supplementary Information.

## Author contributions

B.L. and W.W. conceived and designed the experiments. W.W., J.L. and V.S. directed the project. B.L. performed the flow battery assembly and electrochemical characterizations. B.L. and Z.N. prepared the electrolytes. Z.N. conducted the stability test of the electrolytes. M.V. performed ^67^Zn NMR measurements, DFT calculation and other characterizations. G.L. performed the Raman spectroscopy measurements. B.L., W.W., M.V., G.L. and J.L. co-wrote the manuscript. All authors discussed and analysed the results.

## Additional information

**How to cite this article:** Li, B. *et al*. Ambipolar zinc-polyiodide electrolyte for a high-energy density aqueous redox flow battery. *Nat. Commun.* 6:6303 doi: 10.1038/ncomms7303 (2015).

## Supplementary Material

Supplementary Information Supplementary Figures 1-14, Supplementary Tables 1-2, Supplementary Note 1, Supplementary Methods

## Figures and Tables

**Table 1:** ZIB performance as a function of ZnI_2_ concentration.

ZnI_2_ (M)	CE (%)	VE (%)	EE (%)	OCV (V)	Avg. charge voltage (V)	Avg. discharge voltage (V)
0.5	99.5	91.3	90.9	1.430	1.399	1.265
1.5	99.3	88.7	88.2	1.330	1.343	1.185
2.5	99.0	85.7	84.8	1.285	1.321	1.132
3.5	99.2	76.6	76.0	1.270	1.362	1.066
5.0	96.3	70.4	67.8	1.220	1.330	0.960

CE, coulombic efficiency; EE, energy efficiency; OCV, open-circuit voltage; VE, voltage efficiency.
