# Supplementary material for: Ambipolar zinc-polyiodide electrolyte for a high-energy density aqueous redox flow battery
Source: Nat Commun. 2015 Feb 24;6:6303. doi: 10.1038/ncomms7303 (PMC4346617; doi:10.1038/ncomms7303)
Supplement: Supplementary Information — Supplementary Figures 1-14, Supplementary Tables 1-2, Supplementary Note 1, Supplementary Methods [file ncomms7303-s1.pdf]

Supplementary Figures

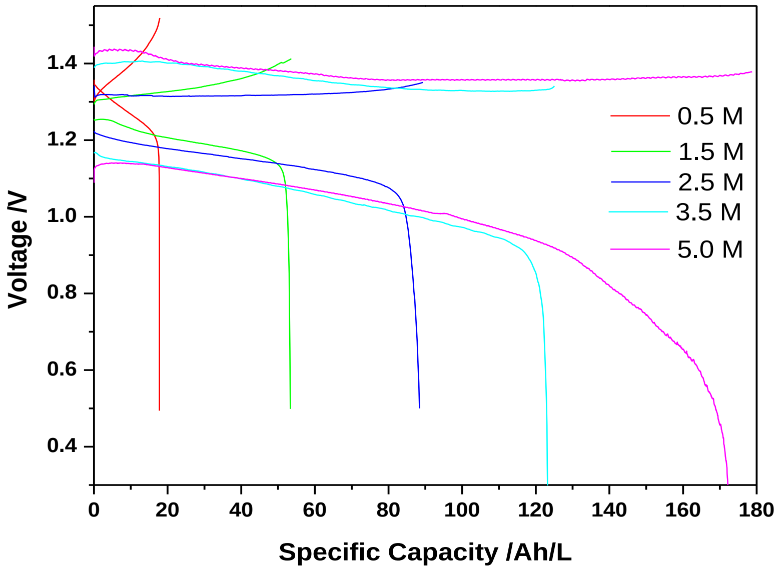

Supplementary Fig. 1. Voltage vs. specific capacity curves at different concentrations of ZnI_2 in water. The current densities are 20 and 5 mA cm^{-2} for 0.5-3.5 M and 5.0 M ZnI_2, respectively.

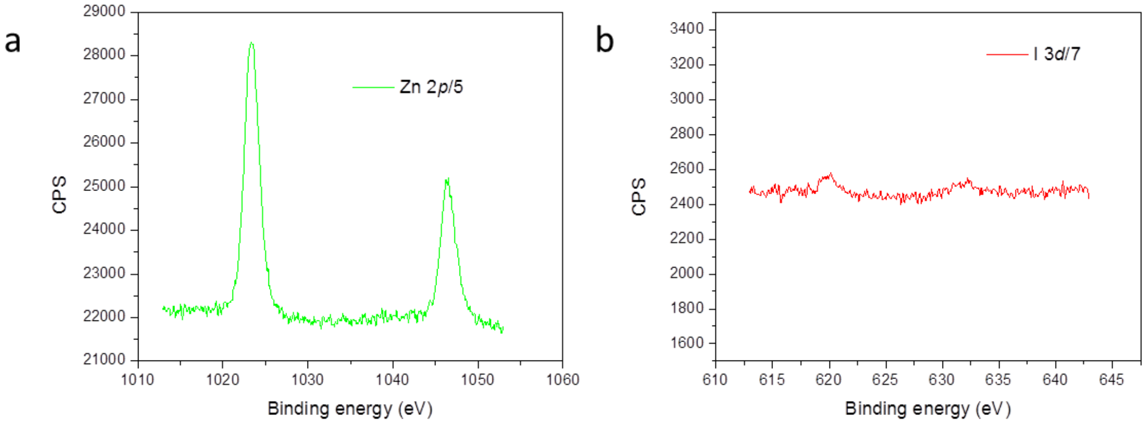

Supplementary Fig. 2. XPS results of the cross sections of the used Nafion membrane in ZIB. (**a**) Zn 2p/5 spectra. (**b**) Iodine 3d/7 spectra.

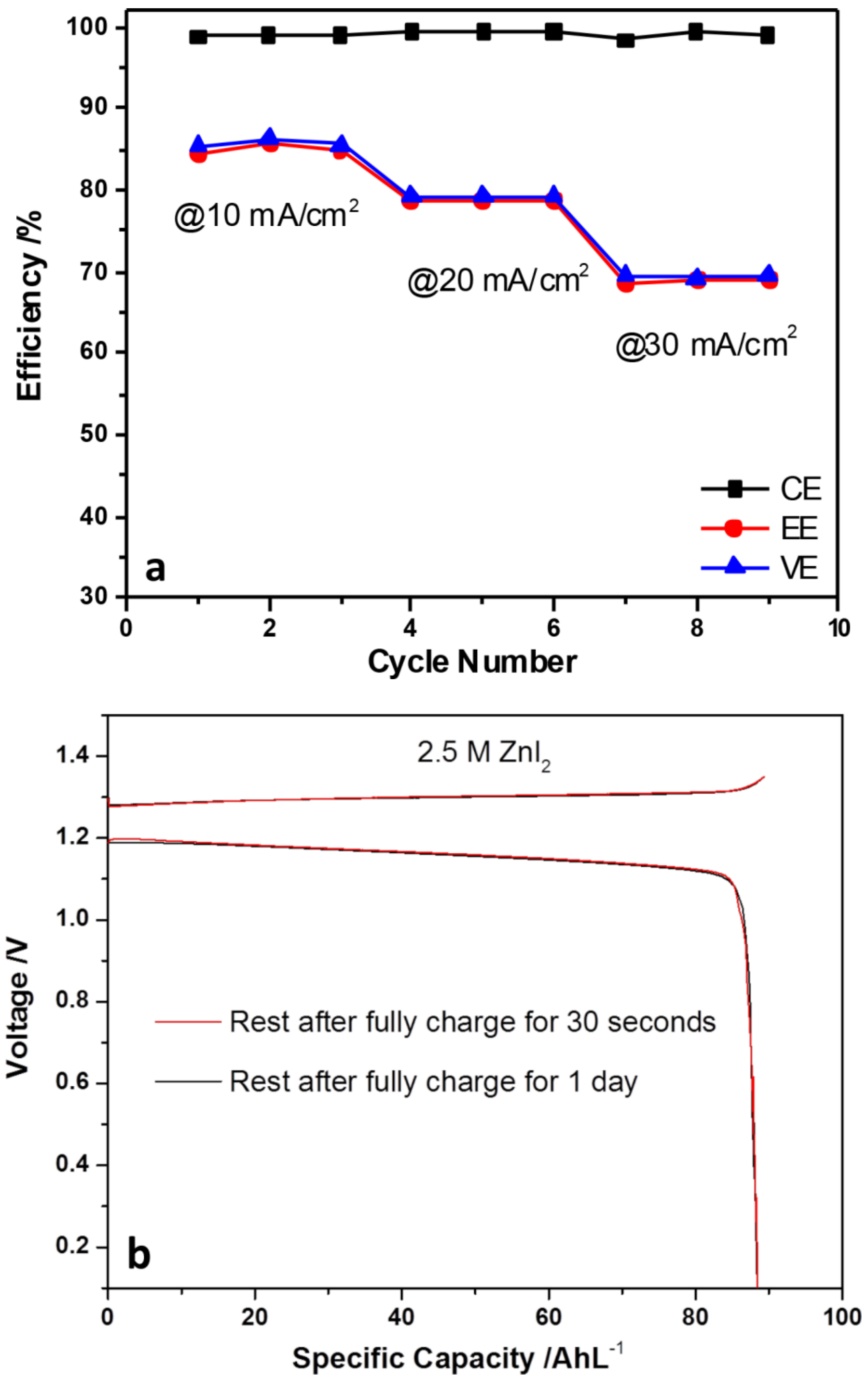

Supplementary Fig. 3. (a) CE, VE and EE values of ZIB cell with 3.5 M ZnI_2 run at different charge/discharge current density, and (b) Voltage profiles of the flow cell test with different rest time.

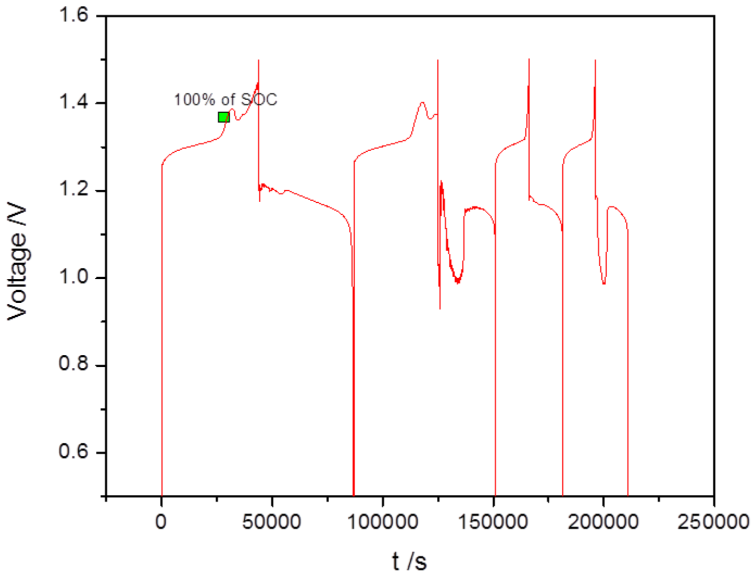

Supplementary Fig. 4. Voltage vs. time curves for the cell with 2.0 M ZnI_2 at the current density of 10 $mAcm^{-2}$.

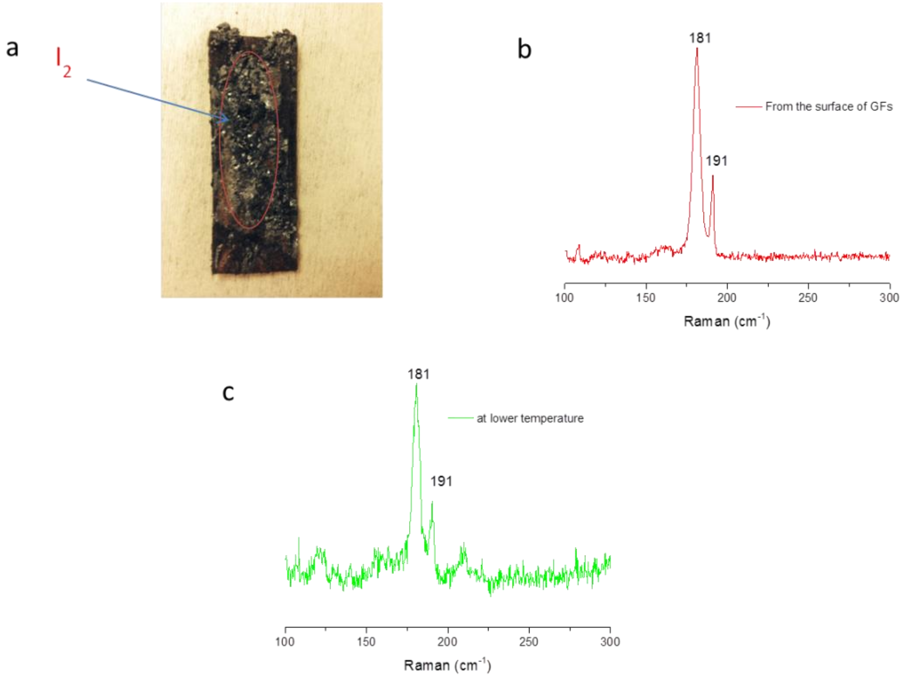

Supplementary Fig. 5. (**a**) GFs at the cathode after charge over 100% SOC (I_2 was found on the surface). (**b**) Raman spectrum of the powders removed from the surfaces of GFs of (a). (**c**) Raman spectrum of the precipitations collected in catholytes at 100% SOC at 0°C. The concentration of original electrolyte ZnI_2 is 2.5 M.

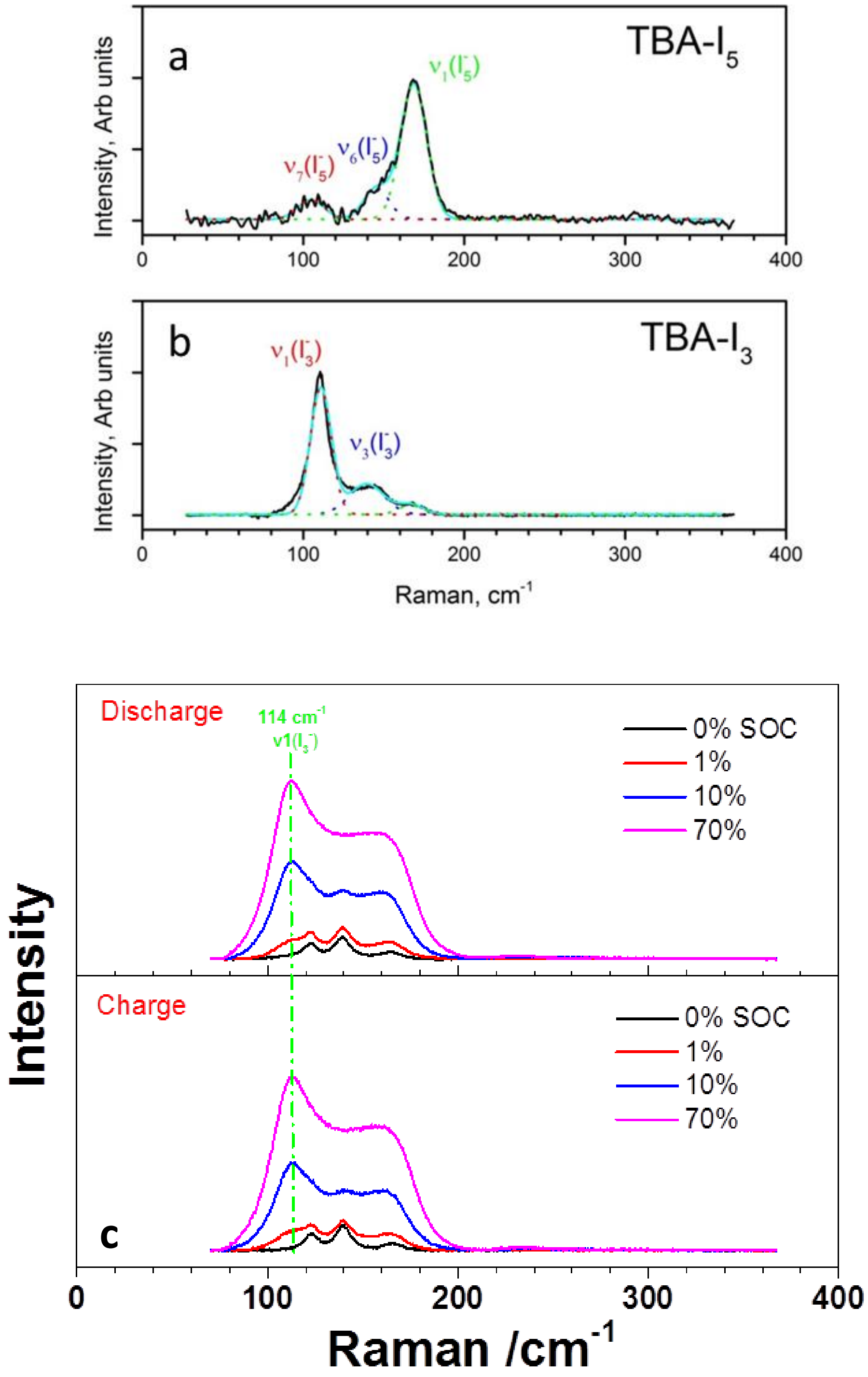

Supplementary Fig. 6. Raman spectra of (**a**) tetrabutylammonium pentaiodide (TBA-I_5), and (**b**) tetrabutylammonium triiodide (TBA-I_3), and (**c**) Raman spectra of catholytes at different state of charges (SOCs) during charge and discharge.

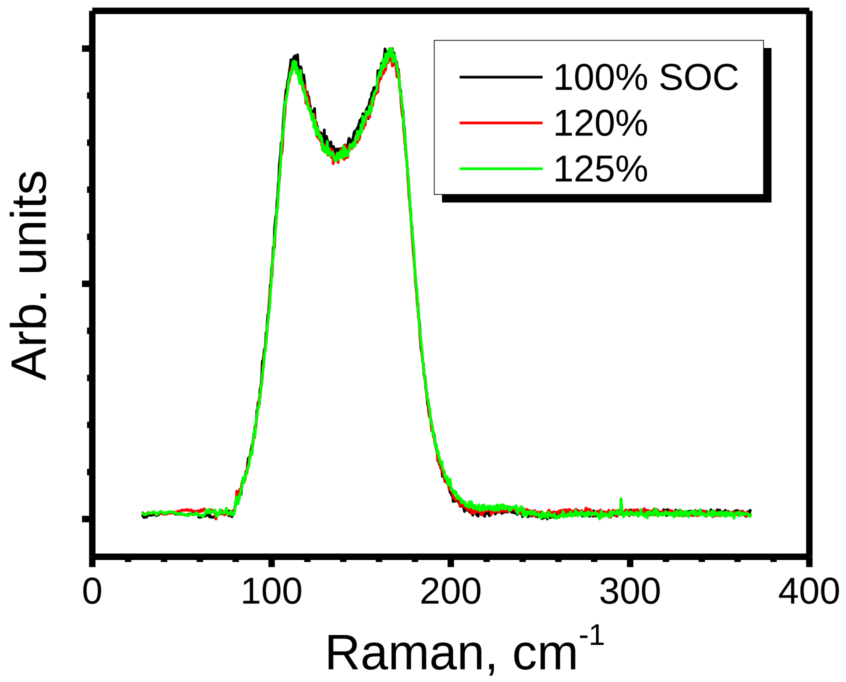

Supplementary Fig. 7. Raman spectra for the catholytes charged to SOCs over 100%.

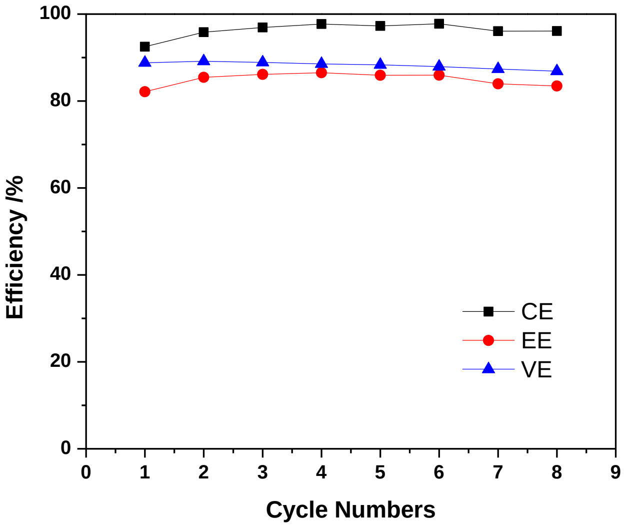

Supplementary Fig. 8. CE, VE and EE for the cell with 3.5 M ZnI_2 operated at the charge/discharge rate of 10 mA cm^{-2} and 50°C with Nafion 115 membranes.

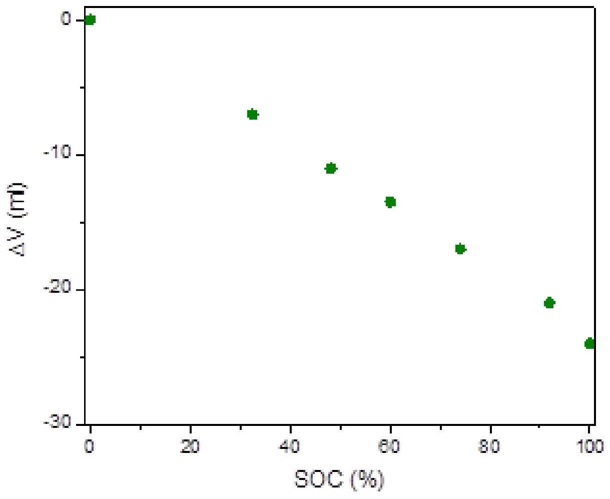

Supplementary Fig. 9. Volume variety of catholytes in 2.5 M ZnI_2 flow cell with SOC. The volume of catholytes at 0% SOC is 90 mL.

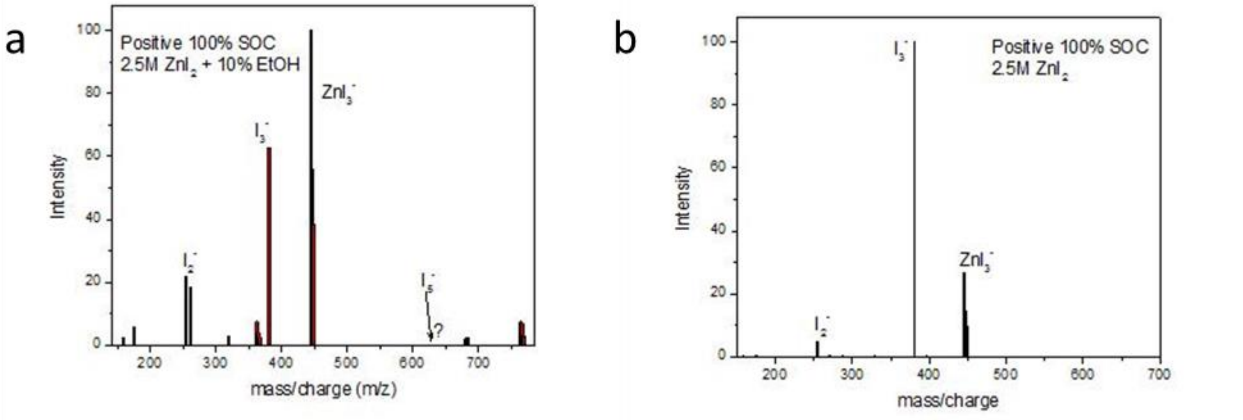

Supplementary Fig. 10. Mass spectrometry analysis of (**a**) pristine and (**b**) EtOH-added catholyte at fully charged condition. The presence of ZnI_3^- and molecular triiodide confirms our NMR and DFT-based analysis. It should be noted that the solutions are diluted more than 1000 times to obtain high resolution spectra. Hence the peak intensity may not represent the respective ion concentration in the electrolyte.

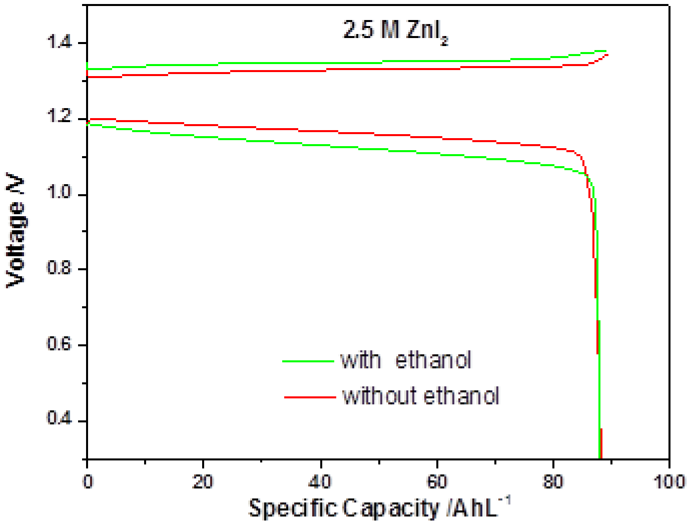

Supplementary Fig. 11. Voltage profiles of a flow cell test on a 2.5M ZnI_2 electrolyte with and without ethanol. The addition of ethanol (10 vol%) will decrease the conductivity of electrolyte, which will then lead to the reduced voltage efficiency (VE) from 88.3% to 84.5% under the same current density (10 mA/cm^2) and operating temperature (25 ^{o}C).

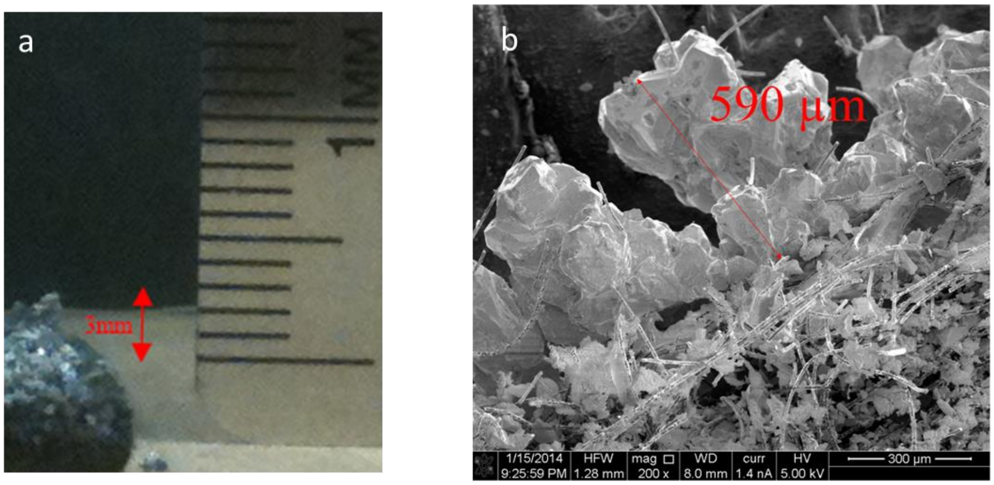

Supplementary Fig. 12. Morphologies of zinc dendrites after charge for the cells with 3.5 M ZnI_2 operated at the current density of 10 mA cm^{-2} (**a**) in the static state and (**b**) the flow rate of 100 mL min^{-1}.

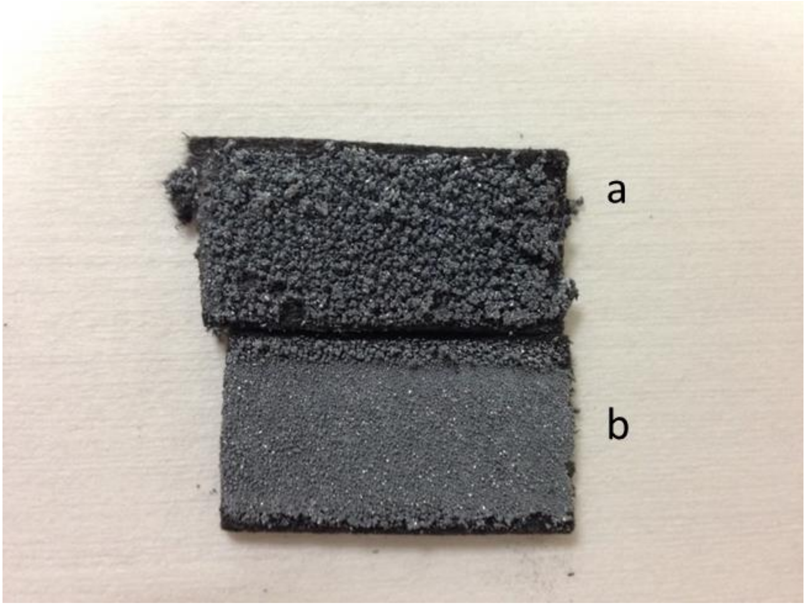

Supplementary Fig. 13. Morphologies of zinc dendrites after charge (**a**) without EtOH and (**b**) with EtOH in the electrolytes.

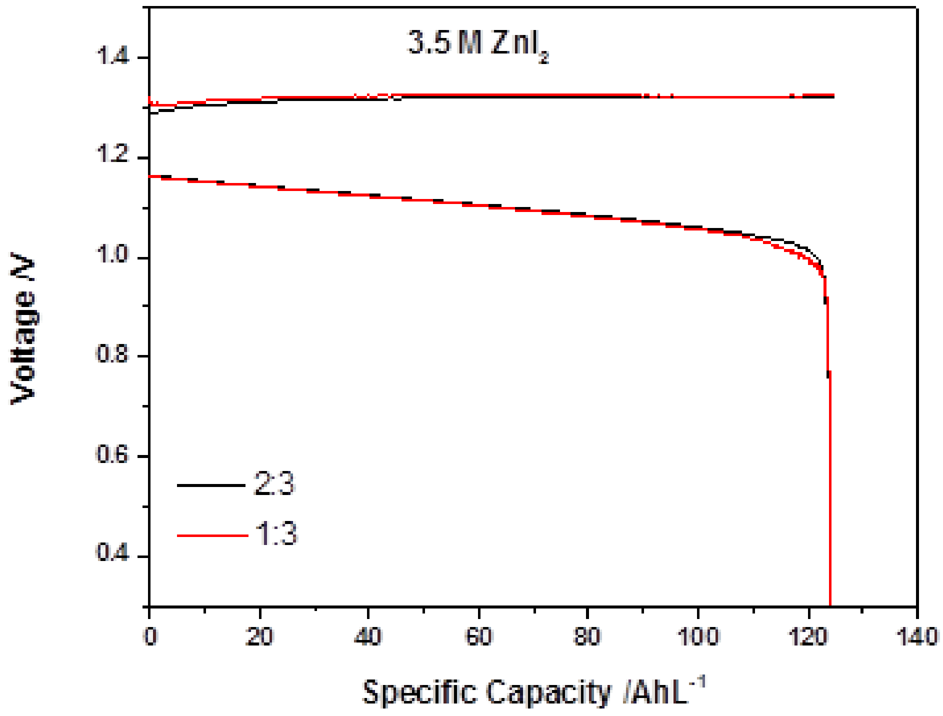

Supplementary Fig. 14. Voltage profile of flow cell tests with different anolyte volumes.

Supplementary Tables

Supplementary Table 1. The other electrochemical performances for ZIB cell with 3.5M ZnI_2 run at different charge/discharge current density.

current density (mA cm^{-2})	OCV (V)	charge voltage (V)	avg. discharge voltage (V)	avg. discharge energy density (Wh L^{-1})
10	1.240	1.298	1.108	141.5
20	1.270	1.362	1.066	136.0
30	1.243	1.416	0.983	125.3

Supplementary Table 2. The stability (off-line) of catholytes at 100% SOC with different concentrations of ZnI_2 at different temperatures in ten days (EG=Ethylene Glycol).

ZnI_2 (M)	vol% EtOH	50°C	25°C	0°C	-10°C	-20°C
3.5	0	stable	stable	unstable	unstable	unstable
	25	stable	stable	stable	stable	stable
	25(EG)	stable	stable	stable	stable	stable
2.5	0	stable	stable	unstable	unstable	unstable
	10	stable	stable	stable	stable	stable

Supplementary Note 1

Estimation of energy density and definition of theoretical capacity

In ZIB systems, during the charge process, zinc ions at the negative side are electroplated onto the surface of the carbon fibers within the graphite felt electrode as the zinc metal. Meanwhile, the zinc ions are also functioning as charge carriers across the membrane. Therefore, the same amount of zinc ions transfer through membranes from positive to negative half-cell to balance the charge due to the negligible transportation of iodide ions through the membrane. The XPS analysis in Supplementary Fig. 2 for a used Nafion membrane after charge shows the presence of zinc metal cations in the water channels. The negligible presence of iodine elements (compared with zinc ion concentration) clearly proves that fully hydrated zinc cation (i.e., $[Zn.6H_2O]^{2+}$) is the main charge transport species during the cycling process. During discharge the reverse process takes place. As a result, the amount of zinc ions will decrease at the positive side and remain constant at the negative side with increasing the state of charge (SOC). In addition to the above analysis, experimental flow cell charge/discharge cyclings with varied anolyte volumes deliver identical energy output. Therefore, it is concluded that the anolyte in a ZIB flow cell does not directly participate in the redox reactions that contribute to the overall cell energy and power delivery. The energy density of a ZIB flow cell is solely dependent on the concentration of the catholyte. Therefore, the volume of effective active species is defined as the volume of cathode electrolyte (n = 1 in Eqn.1). As a result, the theoretical capacity is calculated based on that all the iodides convert into triiodide in catholytes on charge.

To further explain this design, we carried out two different flow cell tests with anolytes/catholytes volume ratio of 1:3 and 2:3, respectively, while the catholyte volumes are the

same 75 mL in both flow cell tests. The concentration of ZnI_2 at 0% SOC at both sides is 3.5 M. As shown in Supplementary Fig. 14, the charge/discharge voltage curves for both cells are nearly identical, confirming our design that the system energy density does not depend on the anolyte volume, but solely on the catholyte, which, on the system level, provide an opportunity to minimize the system volume.

Supplementary Methods

Characterization

In order to confirm the presence of I_3^- and I_5^- in ZIB electrolytes, the commercially available Tetrabutylammonium Triiodide (TBA-I_3, TCI, 99%) and prepared Tetrabutylammonium Pentaiodide were measured using Raman spectroscopy for comparison, respectively. Tetrabutylammonium Pentaiodide (TBA-I_5) solutions were prepared by dissolving TBA-I_3 and I_2 (Sigma-Aldrich, 99.8%) with the ratio of 1:1 in ethanol (Pharmco, ACS gerade) at 95°C for 5 hours. And then the obtained solution was cooled at room temperature and dried for overnight in ambience condition. The black powders (TBA-I_5) were finally obtained. Then Raman spectroscopy measurements were performed on an inverted microscope (Nikon Eclipse Ti) coupled with Raman spectroscopic system (Horiba Jobin Yvon). Red He-Ne laser (632.8 nm) was used as an excitation laser source.

Mass spectrometry (MS) analyses of the samples were performed by electro-spraying the diluted solutions (20x in H_2O / EtOH) from an etched silica capillary emitter (150 micron OD x 20 micron ID) at a flow rate of 1 microliter/minute and floating the metal union at 2.2 kV. The ESI tip was placed at a distance of ~3 mm from a 580 micron ID heated capillary interface (200° C)

to an Exactive mass spectrometer (Thermo Fisher Scientific). In the negative ion mode, polyiodide species dominate the spectrum. All the mass spectra were recorded at a mass resolution of 25000.

The X-ray photoelectron spectroscopy (XPS) analyses were performed using a Kratos Axis Ultra spectrometer (Kratos Analytical, Manchester, UK) equipped with a monochromatized Al X-ray source (10 mA, 15 kV). The Nafion membrane is washed with DI water prior to measurement to remove surface adsorbed electrolyte molecules.

The morphologies of zinc dendrites on the surface of graphite felts (GFs) were characterized by a FE-SEM (JEOLJSM-7600F).
